# Supplementary material for: Are carbon emissions trading and green financial instruments synergistic? -Comprehensive quantitative research based on content analysis
Source: PLoS One. 2024 Mar 7;19(3):e0298601. doi: 10.1371/journal.pone.0298601 (PMC10919601; doi:10.1371/journal.pone.0298601)
Supplement: S1 Data — (ZIP) [file pone.0298601.s001.zip › Data/地级市Z_F工作报告.zip!/║■▒▒/╢⌡╓▌╩╨/└┤╫╘╣1⁄2╓┌║┼í╛┬φ┐╦╩2╛▌═°í┐_2016.docx]

各位代表：

　　现在，我代表市人民政府向大会报告工作，请予审议，并请市政协各位委员和列席人员提出意见。

　　一、“十二五”时期经济社会发展及2015年工作回顾

　　“十二五”时期是我市发展进程中极不平凡的五年。五年来，全市上下以党的十八大精神为指引，在习近平总书记视察湖北、视察鄂州重要指示精神鼓舞下，在省委、省政府和市委的坚强领导下，牢牢把握“三维纲要”，以作贡献、打头阵的精神状态，全面推进“一改两化”，实现了“十二五”圆满收官，经济社会发展取得重大成就。

　　———这五年，是有效应对复杂局势，综合实力跃上新台阶的五年。我们始终坚持抓好发展第一要务，积极应对国际金融危机带来的持续冲击，克难攻坚，砥砺前行，以新作为应对新常态，推动了经济跨越发展。预计2015年，地区生产总值达到730亿元，比“十一五”末（下同）年均增长11.4%。规模以上工业增加值达到382.8亿元，年均增长13%。财政总收入66.2亿元，年均增长16.1%。地方公共财政预算收入47.2亿元，年均增长20.4%。全社会固定资产投资823.69亿元，年均增长22.7%。社会消费品零售总额262.1亿元，年均增长13.8%。外贸出口2.3亿美元，实际利用外资2.28亿美元，年均分别增长11.8%和12.9%。人均生产总值跨越1万美元，标志着鄂州进入新的发展阶段。财政总收入、地方公共财政预算收入、农民人均可支配收入、全社会固定资产投资实现翻一番，为在全省率先全面建成小康社会打下坚实基础。

　　———这五年，是积极调结构转方式，发展质量不断提升的五年。我们始终坚持以质量和效益为中心，加快结构调整，促进转型发展。工业整体实力迈上新台阶。规模以上工业企业达525家，实现主营收入1300亿元，是2010年的两倍。累计实施技术改造项目533个，是“十一五”的2.1倍。企业素质提升。顾地科技在深交所上市，大通物流等23家企业在“新三板”、“四板”挂牌。高能耗、高排放产业比重由2010年的51%下降到30%，高端制造业快速成长，新兴产业比重提高14个百分点，高新技术产业增加值增幅连年居全省前列。现代服务业迅猛发展。电子商务、现代物流快速成长，已经成为新的经济支柱。唯品会、亚马逊、苏宁云商、家乐福、普洛斯、易商、意大利维龙等国内外电商巨头落户葛店开发区，形成大智慧、大物流、大仓储、大市场支撑大电商的产业格局。金融服务实体经济能力提升，2015年底各项贷款余额337亿元，是2010年末的2.3倍。市农村信用社顺利改制成鄂州农村商业银行。

　　———这五年，是咬定发展不放松，重大项目引进建设成果丰硕的五年。我们始终把扩大有效投资作为提升存量、优化增量、加快发展的关键。一批事关鄂州长远发展的重大战略性、支撑性项目落户，特别是建市以来最具全局意义的国际航空物流枢纽项目落户鄂州，是我市“十二五”收官时期的重大喜事。五年累计完成固定资产投资2880亿元，是“十一五”时期的3.4倍。总投资74亿元的鄂州电厂三期工程全面动工，投资过十亿的南都电源、特种汽车、虹润新能源、长煜模具等重大工业项目开工建设。完成交通运输建设投资313亿元，是“十一五”的10倍。汉鄂高速、鄂黄高速、大广南鄂州段、鄂州大道及南互通、城际铁路、新火车站相继建成并开通运行；葛山大道改造、吴楚大道西段即将建成通车，三江港铁路货运枢纽以及综合码头工程建设进展顺利。重大产业和基础设施项目的协同推进，奠定了鄂州未来跨越发展的坚实基础。

　　———这五年，是全面推进沿江滨湖新区开放开发，区域经济竞相发展的五年。我们不断拓展和优化发展空间，创造性地实施“五个一”开发模式，赋予新区市级行政管理权限，大力推动八大新区开放开发。葛店开发区升级为国家级经济技术开发区，产城融合发展步伐加快，综合实力迈上新台阶；鄂州开发区、梧桐湖新区、花湖开发区、红莲湖新区基础设施实现集中连片开发；三江港区成为武汉新港南岸核心港区，公铁水多式联运设施建设全面拉开；鄂城新区高起点规划，建设进展顺利。梁子湖区被列为全国生态文明建设试点，梧桐湖新区被列入全省绿色示范区。2015年八大新区固定资产投资占全市的63.8%，招商引资额占全市的75%，形成了多极支撑、多点发力、竞相发展的开放开发格局。

　　———这五年，是持续改善农村生产生活条件，城乡一体化水平全面提升的五年。我们始终按照“全域鄂州、统筹发展”的思路，推动基础设施一体化和公共服务均等化。城乡一体的交通、供电、供水、广电、通讯等基础设施网络建成，农村卫生、教育、文化等事业全面提升。粮食产量实现“十二连增”，农民收入实现“十二连快”。建成生态农业基地392个，基本实现一村一基地，一大批具有鄂州特色的生态农业品牌兴起。梁子湖区成为全国现代农业示范区，台湾生态农业园、国家有机农产品检验中心落户。农业产业化经营水平全面提升。土地流转率居全省前列。家庭农场、农村专业合作组织等新型农业经营主体快速发展，80%以上农户进入合作服务体系。2015年农产品加工值达到260亿元，是2010年的4.3倍。“互联网+农业”蓬勃发展，特色农产品实现网上销售，鄂州莲子在天津渤交所上市，开启鄂州农产品电子交易新模式。

　　———这五年，是着力提升城市建设管理水平，城乡面貌发生巨大变化的五年。五年来，我们始终以全域规划为先导，坚持建设管理并重，提升城市功能，改善人居环境。累计完成市政基础设施建设投资65亿元，中心城区建成面积达60平方公里。全市城镇化率64.08%，居全省第二。实施城建攻坚计划，三国吴都风光带二期三期、火车站广场、西山公园、橘园公园、博物馆等一批工程建成，西山风景区、莲花山风景区实现提档升级并向市民免费开放，凤凰大桥、主城区综合客运枢纽、雨污分流、洋澜湖湿地公园、市民中心等工程顺利启动实施，葛华水厂投入使用。深入推进“五城同创”，实施城管提能工程，城市形象明显改观，获得全国文明城市提名，顺利通过全国园林城市、省级卫生城复检、全国绿化模范城市核查考评，通过全国旅游标准化城市和省级环保模范城中期评估。在中国社科院全国宜居城市最新排行榜中，鄂州名列第45位，居全省第二。

　　———这五年，是高举改革大旗，发展活力强劲迸发的五年。我们积极承担100多项全国、全省改革试点任务，探索出一批在全省可复制、全国可借鉴的改革经验和鲜活样本。深化行政审批制度改革，实现了全省市州审批项目流程最优、时限最短、收费最低。率先推进“四张清单一张网”建设。围绕打造“一刻钟”服务圈，推进公共服务下沉，120项公共服务事项实现社区办理。深入推进商事制度改革，实施先照后证、注册资本认缴制、“三证合一、一照一码”改革，市场主体总量达6.6万户，五年实现倍增。推动国有资产管理运营制度改革，完成行政事业单位资产清理归集，实行规范化管理、市场化运营。创新投融资体制，积极推动PPP合作、政府购买服务等新模式，设立3亿元产业引导基金、7.5亿元股权投资基金。深入推进农村产权制度改革，农村土地确权登记颁证工作基本完成。扩大农村“五权”抵押融资，发放贷款3.1亿元。全国“多规合一”试点、全国中小城市综合改革试点、全国公立医院改革试点、事业单位分类改革、机关事业单位养老保险制度改革等各项改革试点都取得新成效。

　　———这五年，是建设生态文明，可持续发展能力大幅提升的五年。我们始终坚持把生态文明建设贯穿于发展全过程，积极创建生态市。科学确立主体功能分区，将经济功能主要布局在沿江滨湖新区，梁子湖500平方公里区域全面退出一般工业，在全省率先取消生态区域和农业乡镇招商引资、工业增加值考核。我市被列入全国水生态文明建设试点。加强工业污染源防治，实施105个重点减排工程，火电脱硫脱硝、钢铁烟气脱硫、水泥窑脱硝全部完成。全面禁止秸秆露天焚烧。实施“三边”植树、环梁子湖生态林带、洋澜湖综合治理等工程。涂镇湖、曹家湖实现破堤还湖。实施清洁乡村工程，在全省率先实现城乡一体化垃圾全收集全处理全覆盖，并全面启动全市城乡污水全收集全处理工程，建成污水处理设施221座。建设美丽乡村，完成123个村环境连片整治。4个乡镇获“全国环境优美乡镇”，57个村获“省级生态村”、“全省绿色示范乡村”称号。

　　———这五年，是增进人民福祉，人民群众获得感不断提升的五年。我们始终把保障和改善民生作为政府工作的出发点和落脚点，让人民群众共享发展成果，民生支出占财政支出比重提高到75%。累计投入资金35亿元，连续5年办好“十件实事”。实施精准扶贫，完成整村脱贫阶段性任务。覆盖城乡的社会保险制度基本建立，五项基本社会保险新增扩面14万人。率先在全省对五类特殊困难群众实行兜底保障。率先启动被征地农民社会保障制度。城区低保标准提高到每月460元、农村低保标准提高到每月280元。连续5年提高企业离退休人员养老金待遇。加强村、社区干部工作报酬保障。建设保障性住房3.5万套，启动鄂州职业大学校中村、朱家垴、鄂城水泥厂等棚户区改造。率先在全省完成义务教育均衡发展示范改革，教育信息化、学校标准化等工作步入全国、全省前列。医疗卫生保障体系更加完善，完成中医院、市妇幼保健院改扩建和265个村卫生室标准化建设，市中心医院新门诊大楼投入使用。贯彻计划生育基本国策，出生人口性别比稳步下降。

　　公共文化服务体系进一步完善，文化艺术事业快速发展，全民健身和群众性文化活动蓬勃开展，我市被命名为“中国三国文化之乡”。加强安全生产和食品药品监管，连续6年未发生较大以上安全生产事故，连续14年未发生食品药品安全事件。全面开展平安鄂州建设，社会治安防控体系不断完善，治安和刑事案件发案率持续下降。建立和完善“1+7”社会矛盾纠纷预防化解机制，消除了一批影响社会稳定的矛盾纠纷。推行政府法律顾问和“一村（社区）一律师”制度。“六五”普法圆满完成。民族宗教和谐。国防动员工作不断加强，“双拥”活动取得新成效，海军“鄂州舰”下水。其他各方面工作都取得了新的成绩。

　　各位代表，刚刚过去的2015年，面对错综复杂的宏观经济形势和前所未有的经济下行压力，我们正确认识、适应和引领新常态，积极主动作为，统筹推进“稳、促、调、惠、防”各项工作，经济社会继续保持良好的发展态势。预计全年，全市生产总值增长8.3%，规模以上工业增加值增长7.8%；全社会固定资产投资增长18%，社会消费品零售总额增长13.8%，地方公共财政预算收入增长10.4%，外贸出口增长12.6%，城镇居民人均可支配收入增长9%，农村居民人均可支配收入增长10%，城镇新增就业2.2万人，居民消费价格指数涨幅控制在1.6%。尽管少数经济指标未达到预期目标值，但仍处于较快增长区间，符合经济新常态运行发展规律。

　　与此同时，我们不断加强政府自身建设，巩固党的群众路线教育实践活动成果，扎实开展“三严三实”专题教育，认真执行中央八项规定、省委六条意见和市委实施细则，不断改进工作作风，下大力提高行政效能和服务水平，认真开展“四项专项整治”和“三个专项治理”，落实“三短一简一俭”，“三公”经费比上年下降28%。

　　各位代表，“十二五”时期，我们在复杂严峻形势下取得的这些成绩，得益于市委的正确领导，得益于全市人民齐心协力、奋力拼搏，得益于社会各界的大力支持，在此，我代表市人民政府，向各位人大代表、政协委员，向各民主党派、工商联、无党派人士、人民团体，向驻市人民解放军、武警官兵，向所有参与、支持、关心鄂州建设发展的同志们、朋友们，表示衷心的感谢和崇高的敬意！

　　各位代表，五年的历程令人难忘，五年的成就来之不易。我们深深体会到，推动鄂州科学发展、跨越式发展，必须坚持以经济建设为中心不动摇，牢牢把握发展第一要务，一切从实际出发，把“竞进提质、升级增效”总要求与鄂州实际相结合，厚植发展新优势，把握发展主动权；必须坚持用先进理念引领发展不动摇，坚持科学发展一步到位，牢牢把握“三维纲要”，更加注重发挥市场在资源配置中的决定性作用和更好地发挥政府作用，更加注重保障和改善民生，更加注重生态环境保护与建设；必须坚持打基础、管长远不动摇，应急与谋远相结合，积极谋划推动重大改革发展举措，坚持不懈调结构转方式，系统发挥投资的关键作用、消费的基础作用、出口的促进作用，推动一产业提质、二产业升级、三产业倍增；必须坚持奋发有为、求真务实不动摇，始终保持严实作风，自加压力，主动作为，营造强劲发展气场；必须坚持推进法治政府、责任政府、服务型政府和廉洁政府建设不动摇，全面加强政府自身建设，以法治理念、法治思维推进各项工作，主动接受市人大、市政协监督，为人民办事、对人民负责、向人民报告、让人民满意。

　　同时，我们也清醒地看到，当前全市经济社会发展中还存在一些困难和问题，经济发展总量偏小、层次偏低，财政增收压力大，创新能力不强，开放水平不高，长期积累的供给侧结构性矛盾集中，等等。对于这些问题，我们不回避、不懈怠，一定以高度负责的精神，采取切实有力的措施，认真加以解决。

　　二、“十三五”时期经济社会发展的主要目标和任务

　　“十三五”时期，将是鄂州在新的历史起点上，大有可为的黄金机遇期。我们要抢抓国家加快推进中部崛起、长江经济带、长江中游城市群建设等历史机遇，以强烈的事业心和高度的责任感，奋力开创“十三五”发展新局面。根据《中共鄂州市委关于制定全市国民经济和社会发展第十三个五年规划的建议》，市政府编制了《鄂州市国民经济和社会发展第十三个五年规划纲要（草案）》，提请大会一并审议。

　　“十三五”发展的指导思想是：高举中国特色社会主义伟大旗帜，全面贯彻党的十八大和十八届三中、四中、五中全会精神，以马克思列宁主义、毛泽东思想、邓小平理论、“三个代表”重要思想、科学发展观为指导，深入贯彻习近平总书记系列重要讲话精神，全面落实“四个全面”战略布局，统筹推进经济建设、政治建设、文化建设、社会建设、生态文明建设和党的建设，深入贯彻落实五大发展理念，牢牢把握“三维纲要”和“竞进提质、升级增效、以质为帅、量质兼取”工作方针，坚持发展第一要务，以率先全面建成更高标准小康社会和加快城市现代化进程为主题，以建设国际航空都市区为主战场，以转方式、调结构为主线，实施创新驱动、协调带动、绿色引领、开放先导、共享保障战略，不断创造鄂州速度、鄂州质量、鄂州效率、鄂州标准，在湖北“建成支点、走在前列”征程中作贡献、打头阵。

　　今后五年全市经济社会发展总体目标是：综合竞争力进一步增强，生产总值年均增长9%左右，到2020年力争达到1200亿元，人均GDP突破10万元，全社会固定资产投资年均增长15%，公共财政预算收入年均增长12%；人民生活水平进一步提高，城乡居民人均可支配收入与经济同步增长，城乡公共服务均衡发展，贫困人口全部脱贫；社会文明程度进一步提升，市民科学文化素养和健康文明素质普遍增强；生态环境质量进一步改善，国家生态文明试验区创建取得突破，环境质量明显改善，全面完成省定节能减排目标；城市治理现代化进一步加快，城市宜居性增强，法治鄂州建设深入推进，各项改革任务如期完成。

　　今后五年，我们要以“创新、协调、绿色、开放、共享”五大理念引领发展，奋力推进经济社会发展实现新的跨越。

　　（一）坚持创新发展，建设活力城市。实施创新驱动战略，让创新贯穿经济社会发展全过程，增强科技创新力，把创新摆在鄂州发展全局的核心位置，提高创新服务水平。加快引进创新步伐，强化企业创新主体地位，形成一批创新型领军企业。培育发展新动力，发挥消费的基础作用、投资的关键作用、出口的促进作用，加快实现发展动力转换。拓展发展新空间，构建发展新格局，实施“互联网+鄂州”计划，推动沿江城市连绵带建设。全力推动产业转型升级，突破性发展现代服务业，加速发展先进制造业，大力推进农业现代化。建设全面深化改革示范区，深化行政管理体制改革，深化要素配置市场化改革，深化财税管理体制改革。

　　（二）坚持协调发展，建设品质城市。实施协调带动战略，进一步增强发展的平衡性、协同性、整体性。提高城市发展质量，提升城市规划、建设和管理水平。提高美丽乡村建设质量，加大传统村落民居和历史文化名村名镇保护力度，集中连片规划建设美丽乡村。提高精神文明建设质量，加强法治鄂州建设，开展社会主义核心价值观教育，打响鄂州文化品牌，突破性发展文化产业，丰富城乡文体生活，建设文化强市。提高军民融合发展水平。

　　（三）坚持绿色发展，建设生态城市。实施绿色引领战略，建设国家生态文明试验区和优质生态产品生产基地，打造湖北水生态安全屏障。加快生态文明建设，全域开展生态文明创建，重点打造梁子湖生态文明示范区。加快绿色发展进程，推行生产生活方式绿色化，完善绿色发展机制，建立绿色财税机制，构建绿色金融体制，建立绿色考核机制。

　　（四）坚持开放发展，建设国际城市。充分发挥国际航空物流枢纽项目对城市功能、产业升级、要素集聚的引领功能，加快推进航空都市区基础设施建设，建成立足长江、辐射全国、面向全球的航空物流枢纽中心。构建航空、水运、铁路、公路有机衔接的多式联运体系，加快“一心两翼”现代化大港口建设，打造武汉新港南岸核心港区，建设区域陆路交通枢纽。提高对外开放水平，学习借鉴上海自贸区经验，推行自贸区体制机制，打造对外开放平台，加快优进优出步伐，深度融入长江中游城市群。实施开放先导战略，建设长江中游重要节点城市，打造内陆开放门户。

　　（五）坚持共享发展，建设幸福城市。实施共享保障战略，切实保障改善民生，让人民群众有更多获得感、幸福感。实施创业就业工程，实行更加积极的创业就业政策，完善创业就业服务体系，持续增加居民收入。实行教育现代化工程，推进教育优质均衡发展，构建现代教育体系。实施全民社保工程，推进全民参保计划，完善社会救助体系，发展社会福利事业，改善居民住房条件。实施健康鄂州工程，深化医药卫生体制改革，提高医疗服务质量和水平，增强公共卫生服务能力，促进人口均衡发展。实施脱贫攻坚工程，落实精准扶贫政策，完善扶贫工作机制，确保2019年贫困人口全部脱贫销号、贫困村全部脱贫出列。三、年工作安排

　　2016年是“十三五”开局之年，是全面建成小康社会决胜阶段的首战之年。做好今年工作，意义深远，责任重大。面对发展新常态，我们要清醒地看到，新的一年宏观形势依然严峻，经济下行压力继续加大。必须以竞进的精神状态，克难攻坚，抢抓机遇，积极做好各项工作，确保“十三五”开好局、起好步。

　　今年全市经济社会发展主要预期目标是：地区生产总值增长9%，规模以上工业增加值增长8.5%；全社会固定资产投资增长16%以上；地方公共财政预算收入增长10%，社会消费品零售总额增长13.5%以上，外贸出口增长10%，居民收入与经济同步增长，城镇新增就业1.8万人，完成省定节能减排目标。

　　今年重点抓好以下八个方面工作：

　　（一）着力调结构转方式，推进经济转型升级。依靠创新驱动，强化先进制造业的主导作用、新兴产业的引领作用、现代服务业的支撑作用，着力稳增长调结构，强力推进产业升级。一是再造工业发展新优势。坚持改造传统产业与培育新兴产业并重，加强传统企业技术改造，推进工业化与信息化融合。启动实施“百家企业技改行动计划”，全面提升技术装备、经营管理、品种质量水平，培育一批高端拳头产品，提升全要素生产效率，促进传统工业向信息化、智能化、绿色化迈进。把新兴产业培育作为经济工作的主战场，集中力量支持生物医药、电子信息、新材料等产业形成集群优势。发挥产业基金对新兴产业培育、高新项目引进的撬动作用，支持北斗导航、物联网、机器人、高端智能装备、新能源等项目快速成长。加快电厂三期、南都电源、特种汽车、虹润新能源等重大项目进展，尽快形成百亿产业规模。实施“中小企业成长工程”，确保亿元骨干企业增加15家，规上企业新增50家。实施“推动企业上市计划”，吸引产业基金以“股权+债权”的方式，支持企业上市、挂牌，扩大直接融资。二是推进现代服务业做大做强。把现代服务业作为扩大经济总量的引爆点，着眼于千亿产业目标，高起点谋划重大推进政策、发展规划和承载平台。加快推进葛店中部电子商务基地建设，争取开展跨境电子商务试点。推动电子商务在各领域的推广应用，实现电商产业集聚和园区联动发展，力争全市电子商务交易额达到150亿元。支持南山临港新城一期、东方甩挂联盟、三江港公铁物流等项目建设。提高对外贸易水平，力争出口实绩企业达到60家。投入5000万元注入文化产业基金，培育发展文化产业。推进全国旅游标准化示范城市创建，打造一批山水乡愁和历史文化旅游精品。三是增强创新驱动和创业发展能力。强化企业创新主体作用，支持开展产学研合作，出台以奖代补政策，支持引进、自建国家省级重点实验室、工程技术研究中心、企业技术中心、院士和博士后工作站，力争全年高新企业增加10家以上。实施质量强市战略，支持国家省级质量检测中心建设。构建一批全要素、开放式的众创空间，支持葛店科技加速器、鄂州开发区创业咖啡等平台健康发展，运用“孵化器+风险投资”、“加速器+股权投资”、“众创、众包、众扶、众筹空间+天使基金”模式，扶持一批创新型小微企业成长，加快形成新的产业业态和增长点。四是大力推进供给侧结构性改革。认真落实去产能、去库存、去杠杆、降成本、补短板改革任务，谋划实施“供给侧改革鄂州行动计划”。继续实行服务企业“直通车制度”，帮助企业加强产业链对接，搭建银企对接桥梁，提高财政间歇资金使用效率和针对性。新增6000万元财政资金，支持企业技术改造、节能减排和补充保证保险贷款基金等，支持实体经济发展。开展“降低企业成本行动”，落实国家减税降费政策，扩大直供电交易试点，帮助企业降成本、增效益。最大限度减少投资前置审批事项，促进投资便利化。依靠市场力量，推动冶金、建材、矿山等企业兼并重组，淘汰一批污染严重、浪费资源、安全保障难度大、丧失自我更新发展能力的落后企业。

　　（二）深入推进改革攻坚，增创体制机制新优势。突出问题导向和目标导向，全面推进各项改革，增强发展动力和活力。一是深化经济体制改革。加快建立符合市场化要求、促进转型发展的投融资、财税、国有资产管理体制机制。综合运用PPP模式，推动重大项目建设融资。继续推进营改增试点，将建筑业、房地产业、金融业和生活服务业纳入营改增范围。按照公共财政要求，优化支出结构，加强对生态建设、社会保障、精准扶贫、基层治理的支出保障。建立完善以管资本为主的国有资产监管新体制，推进区级行政事业单位资产归集，推进经营性国有资产集中统一监管，启动文体旅游和自然资源资产归集、授权经营工作。二是深化行政管理体制改革。进一步简政放权，完善“四张清单一张网”，推进行政审批事项全流程、并联式网上审批和电子监察全覆盖，建立健全事中事后监管体系。进一步推进商事制度改革，推动工商登记全程电子化以及电子营业执照试点。开展综合监管行政执法改革试点。认真做好行政审批事项的承接落实和取消下放工作，提升新区承接市级职权的能力水平，提高新区行政运转效能和服务效率。三是深化农村改革。推进农村基层交易平台建设，实现信息对接。开展赋予农民对集体资产股份占有权、收益权试点。推进新型农民专业合作社联社试点，推动金融与农民专业合作社深度融合。在“三权分置”的基础上，通过土地经营权入股等方式发展多种形式规模经营。推进国有林场改革和供销社综合改革。四是深化社会管理体制改革。推进社会治理精细化，完善社会治理责任体系。加强矛盾纠纷调处平台建设，健全完善“1+N”多元社会矛盾纠纷预防化解机制。推动城乡社区治理体制机制创新，加强社区软硬设施建设，支持基层社会组织发展，增强社区和基层社会组织的自我服务、自我管理能力。同时，加快全国中小城市综合改革试点、“多规合一”试点和不动产统一登记制度改革等各项改革工作。

　　（三）加快推进新区建设，开创开放发展新局面。抢抓国家长江经济带、长江中游城市群战略机遇，把航空都市区和新区建设作为主阵地、主战场。一是全力推进航空都市区建设。省委、省政府要求2020年在鄂州实现大飞机起降，任务光荣而又艰巨。按照要件申报、机场核准、工程建设、竣工验收等时间节点，倒排工作量，明确任务清单，落实责任主体，实行挂图作战，并行推进各项工作。高起点编制完成航空都市区总体规划和产业规划，设立200亿元航空产业发展基金，启动拆迁安置、道路交通等配套设施建设，开展临空产业的引进和导入。推进临空金融商务区建设，引进各类金融机构和多层次资本市场主体入驻，打造长江中游“金融港”和“基金小镇”。二是完善新区基础设施。以人为本理念指导八大新区建设发展，把新区建成独立成篇的现代化宜居宜业新城。加强新区教育、医疗、文化娱乐、超市、集贸市场等消费设施建设，让新区引得进人、留得住人。启动建设葛店新高中、梧桐湖医院、葛华客运站。继续高强度进行基础建设投入，打通制约项目落地“最后一公里”，提升产业承载力。抢抓国家放松银根，实行低利率、长周期的政策性融资机遇，扩大建设性融资规模。三是加快新区产业发展。坚定不移走产城融合路子，狠抓新区产业成长。葛店开发区重点发展生物医药、高端制造、电子商务、现代物流等产业；鄂州开发区重点发展节能环保、智能制造、新型建材等产业；鄂城新区和花湖开发区重点发展临空经济；三江港和红莲湖新区重点发展港口物流、休闲旅游等产业；梧桐湖新区和梁子湖生态文明示范区重点发展生态农业、生态旅游、文化创意等产业。立足新区产业定位，加强招商引资，推动项目向新区集中，引进一批投资过10亿元的大项目落户，打造沿江产业集聚区。

　　（四）大力发展生态农业，加快建设美丽乡村。始终把“三农”工作作为重中之重，加快转变农业发展方式，促进农业增效、农民增收、农村繁荣。一是提升生态农业发展水平。坚持生态农业的发展方向不动摇，促进种养加一体、一二三产业融合发展。落实粮食安全责任制。加快推进种植规模化、生产标准化、产品特色化、农业机械化、营销品牌化，实现农业提质增效。推进台湾生态农业园、省级现代农业示范基地等农业示范园区建设。大力发展农产品加工业，培育龙头企业。围绕现代农业经营体系建设，做大做强新型农业主体。加大职业农民培育力度。实施品牌兴农战略，优化“三品一标”农产品认定认证。二是全面加强农村基础设施建设。改善农村生产条件，提高粮食综合生产能力。加快推进城乡道路交通网络建设维护，安排专项资金扶持农村公路的养管护工作。推进水利基础设施建设，实施花马湖灌区节水配套改造、新港河道整治、民信闸除险加固、樊口电排二站等工程。大力实施农村电网改造，提高产业化基地、农村新社区台区供电能力。完善农村安全饮水体系，完成雨台山水厂扩建，建成太和水厂，新建城东水厂和城南自来水加压站，提高城乡供水能力和质量。三是大力发展“互联网+农业”。加强农村信息网络建设，推进“宽带鄂州”建设，完成光纤进村入户工程，为“互联网+”融入农业农村发展创造条件。整合全市涉农信息资源，搭建集生态农业信息传播、优质农产品展示和网络营销于一体的电商平台，支持龙头企业、种养大户入驻平台。积极与国内知名电商合作，推动农业从传统营销业态转变为“线上线下相结合、前台后台一体化”的电子商务模式，全域推进电商进农村，培育一批“淘宝村”、“淘宝户”。四是提升美丽乡村建设水平。按照生产、生活、生态相和谐理念，不改变传统村落形态，美化传统民居环境，加强乡村绿化，因地制宜、一村一特色。注重发挥农民主体作用，不搞违背农村生产生活特点和农民意愿的大拆大建。以村村秀美、户户整洁为目标，整体规划、整村推进农村环境综合整治，改善农村人居面貌。

　　（五）提升城市功能品质，营造优美宜居环境。坚持“五个统筹”，完善城市功能设施，提高城市治理能力，建设生态宜居文明城市。一是加强城市功能建设。强力推进城建“重大项目建设年”活动，开工建设鄂咸高速、重载车专用通道，完成三江港区综合码头一期、五丈港港区综合码头、吴楚大道东段主体工程。启动南互通至铁山、东沟至铁山一级公路建设，三江大道与吴楚大道实现对接。加快推进凤凰大桥、市民中心、东洋澜湖湿地公园、吴都中央生态廊道二期等工程建设，启动文化中心、体育中心、天龙山公园、江滩公园四期、植物园、动物园等重点工程。统筹城市地下和地上设施建设，推进“海绵城市”和综合地下城市管廊建设，完成主城区雨污分流，全部封堵入洋澜湖排污口。推进老旧城区、城际铁路沿线棚户区破旧危房拆迁改造，完成1.5万套棚户区和危房改造任务。探索建立多形式、多元化城市建设投融资体系，力争全年全市基础设施投资突破200亿元，主城区完成投资20亿元以上。二是深入开展城管能力提升工程。继续以“五城同创”为载体，推进城乡环境综合整治，整治范围向城中村、城郊村、老城区、背街小巷和农村延伸，向各区、开发区、中心镇覆盖。实行重点专项治理与日常精细管理相结合，开展渣土抛撒、工地扬尘、油烟噪声、乱搭乱建、出店经营、交通违法等专项整治，形成条块结合的长效管控机制。认真解决好物业管理纠纷、集贸市场脏乱差等一系列问题，加大停车场、微循环等城市交通疏导场所建设。完善全域公共交通体系，推动城市公交向新区和乡镇延伸，优化班次线路，逐步实现全市通勤一体化。重点加强大冶至武汉鄂州路段治超工作。推进“智慧城管”建设，运用互联网和大数据技术，建立智能化城市管理平台，提升管理水平。三是推进农村人口市民化。深化户籍制度改革，推动户籍制度改革和居住证双落地，推进农民工市民化，先期重点推进长期在城市居住、有相对固定工作的农民工有序市民化，实现安居乐业。把房地产去库存与城市化工作结合起来，把货币化安置和拆迁还建结合起来，对被征地拆迁农民，按城市化一步到位的要求，统筹解决其户籍、住房、社保、医保、养老、子女就学等问题。深化基本公共服务供给制度改革，促进有条件有意愿的农民工及家属在城镇有序落户，平等享受基本公共服务。

　　（六）坚持绿色低碳发展，建设生态文明示范区。把生态文明建设作为转变发展方式的突破口和着力点，完善绿色发展机制，加快绿色发展进程。一是推进全域生态建设。以创建生态市为契机，全域推进生态文明试验区建设。依托梁子湖综合防洪一期工程等重大项目，推进全国水生态文明试点建设，集中连片实施山水田林湖生态保护和修复工程，推进湖泊生态治理，对湖泊流域实行用途管制。完成花马湖水系生态保护规划，启动青天湖、五四湖、葛店南部区域湖泊生态恢复工程。开展生态乡镇创建，建设一批国家级、省级和市级生态乡（镇）村。对全市百年以上古树名木实行编号保护。全面推进“绿满鄂州”建设。二是加强生态环境整治。统筹城乡生态环境整治工作，建立政府、企业、公众共治体系。实行污染源控制和污染物治理两手抓，加强工农业生产污染源监管，实施一批节能减排工程，严控农村面源污染。加快实施城乡一体的污水收集处理体系建设，推进红莲湖污水处理厂和葛店三王污水处理厂建设，鄂钢生产生活污水全部进入城市污水管网。加强大气污染防治，强化秸秆焚烧管控工作，推广秸秆资源化综合利用，淘汰所有“黄标车”，城区禁止使用燃煤锅炉，推行“煤改气”。三是探索绿色发展促进机制。推动建立自然资源资产产权制度，探索绿色资产证券化、绿色信贷融资模式，积极开展资源和环境责任审计试点。划定生态红线、基本农田红线和城市边界线，城区实行“退二进三”。启动生态补偿机制，从今年开始，每年市财政转移支付补助梁子湖区1500万元，补助15个农业乡镇各50万元。抓好资源节约集约利用，实行能效、建设用地强度“双控”措施，推进城镇低效用地再开发和工矿废弃地再利用。开展梧桐湖新区绿色建筑示范区建设。大力倡导全民节能环保意识，形成绿色低碳社会风尚。

　　（七）加强保障和改善民生，共享改革发展成果。建设幸福城市，我们要多谋民生之利，多解民生之忧，增进人民群众福祉。一是扩大城乡就业。实施创业引领计划，统筹推进高校毕业生、农村转移劳动力、城镇困难人员等群体就业。优化小额担保贷款发放办法，促进创业带动就业。加强就业指导服务，开展多种形式就业技能培训，提高劳动者就业能力。二是加强民生保障。完善特困群体兜底保障制度，适当提高农村养老保险、老年补贴标准。统一城乡医保险种“三个目录”，提高医保报销比例。抓好农村福利院提档升级工作，新增城镇社区养老、农村老年人互助养老机构22处，大力发展“医养结合”养老模式，启动民政服务中心、残疾人康复托养中心建设。推进机关事业单位养老保险制度改革。建立农民工工资保障制度。三是发展社会事业。推进教育信息化试点改革，建设市中等职业教育中心，新建城东九年一贯制学校，启动市二中迁建工程，建设一批普惠性幼儿园，加快推进华中师范大学鄂州校区建设，筹资8000万元改善薄弱学校办学条件，办人民满意教育。深化医药卫生体制改革，加强基层医疗队伍建设，完善卫生计生服务设施，提高医疗卫生保障水平，启动儿童医院建设，实施全面“两孩”政策。加快文化体育事业发展，广泛开展全民阅读、全民健身、群众性文化活动，完善城乡公共文化服务体系，加强吴王城遗址等历史文物保护。四是打好精准扶贫攻坚战。认真落实国家、省和市精准扶贫各项政策，安排财政扶贫资金1.1亿元，按“六个精准”要求，锁定对象，精准施策。支持有一定能力的贫困家庭发展特色生产和转移就业。贫困人口医保报销比例提高10%，并提供医疗救助，特困残障群体一律实行兜底保障，确保年底45%的贫困村出列、45%以上贫困人口脱贫。完成农村危房改造任务。五是增强社会安全感和满意度。完善城乡网格管理。创新完善立体化治安防控体系，深入开展平安创建工作。抓好特殊人群收治、管护。加强社区矫正工作。强化安全生产责任制，提高安全隐患排查治理体系建设水平。加强产品质量和食品药品安全监管。畅通群众诉求反映渠道，着力化解社会矛盾。强化应急管理，提高防灾减灾、保障公共安全和处置突发事件能力。深入推进法治鄂州建设。继续推进国防动员和“双拥”共建等工作，进一步巩固军政军民关系。

　　（八）加强政府自身建设，打造人民满意政府。面对改革发展的新形势，人民群众的新期盼，我们必须加强政府自身建设，牢记工作职责，不负人民重托。推进依法行政。加快法治政府建设，强化法治意识，严格按照法定权限和程序履行职责、行使权力。启动“七五”普法，依法制定行政规章，依法依规推动改革发展。健全依法决策机制，全面推进决策公开、执行公开、管理公开、服务公开、结果公开，让权力在阳光下运行。完善行政处罚自由裁量权基准制度，创新行政复议办案机制。自觉接受市人大及其常委会的法律监督和工作监督，严格执行市人大常委会决定，自觉接受市政协的民主监督，认真办理人大代表建议和政协提案，广泛听取各民主党派、工商联、无党派人士和社会各界意见，自觉接受各方面的监督。提升行政效能。集中精力谋大事、抓发展。加强政风建设，崇尚实干，力戒空谈，雷厉风行开展工作。加强效能监察和问责，对各项工作实行项目化、目标化、责任化管理，严格绩效考评，切实解决不作为、慢作为等问题，提高工作执行力和效率。巩固群众路线教育实践活动和“三严三实”专题教育成果，实现服务群众、解决问题的常态化，从今年开始，我们改变过去集中力量办“十件实事”的做法，把为人民办实事寓于日常工作之中，让人民群众时刻都能感受到政府服务的改善和效能的提升。强化责任担当。把思想和精力集中到谋事创业上，把功夫下在狠抓落实上。要以作贡献、打头阵的精神状态，紧盯重点和难点，想对策、下功夫、求突破。加强新知识学习，加强新政策研究，不断总结、创新、提高，用新思维、新办法适应新常态，确保各项工作实现新的突破。坚持廉洁从政。坚持“力度统一论”，认真贯彻《中国共产党廉洁自律准则》、《中国共产党纪律处分条例》和《行政机关公务员处分条例》，落实党风廉政建设主体责任，坚持道德高线，守住法纪底线。健全政府系统惩治和预防腐败体系，加强对财政资金分配使用、项目招投标、工程建设、土地出让、政府采购等重点领域和环节的监管，厉行勤俭节约，反对铺张浪费，严控“三公”经费。强化行政监察和审计监督，推行重大决策和规范性文件廉洁性评估，从源头上预防和治理腐败，营造干净干事、风清气正的良好氛围，努力做到干部清正、政府清廉、政治清明。

　　各位代表，催人奋进的“十三五”新征程已经开启，新的一年，让我们在省委、省政府和市委的坚强领导下，同心同德，锐意进取，扎实工作，为在全省率先全面建成更高标准小康社会、加快城市现代化进程而努力奋斗！
